# Active Visual Art Therapy in the General Hospital: Facts and Challenges from an Ethical Perspective

**DOI:** 10.3390/ijerph22020316

**Published:** 2025-02-19

**Authors:** Valentina Martinelli, Estella Linda Luisa Lumer, Laura Fusar Poli, Matteo Chiappedi, Pierluigi Politi

**Affiliations:** 1 General Surgery Unit 2, IRCCS Policlinico San Matteo, 27100 Pavia, Italy; 2 Harvey Medical Course, Department of Molecular Medicine, University of Pavia, 27100 Pavia, Italy; estellalindalu.lumer01@universitadipavia.it; 3 Department of Brain and Behavioral Sciences, University of Pavia, 27100 Pavia, Italy; laura.fusarpoli@unipv.it (L.F.P.); pierluigi.politi@unipv.it (P.P.); 4 Istituto Dosso Verde, 27100 Pavia, Italy; matteo_chiappedi@asst-pavia.it

**Keywords:** visual art therapy, ethics, general hospital, mental health, physical health

## Abstract

For decades, art in its many forms has been used to improve patients’ quality of life and mental health. A growing amount of literature has shown the effectiveness of active visual art therapy (AVAT) on different patient outcomes and highlighted the need for international collaboration and harmonization of research methods. Evidence regarding AVAT inside the general hospital is still limited. This context poses unique challenges in terms of feasibility, heterogeneity, settings, and type of participants, together with significant ethical implications in terms of humanization of care. This narrative review aimed to report the available data on the effectiveness of visual art therapy in the general hospital and discuss them through the lens of the key bioethical principles of autonomy, beneficence, non-maleficence, and justice introduced by Childress and Beauchamp. Current evidence supports the effectiveness of AVAT on children and adult inpatients’ outcomes, particularly in the areas of pain control, anxiety, and depression, therefore supporting the individual’s autonomy and beneficence. With regard to justice and equity, AVAT proved to be a safe and cost-effective adjunct intervention to medical management inside the hospital. A more in-depth understanding of the ethical aspects implied in using AVAT in the general hospital may add a further contribution to the implementation of art interventions in patient-centered care.

## 1. Introduction

For decades, art in its many forms has been used to improve people’s quality of life and mental health. Poetry, theater, music, and visual arts have been employed to process traumatic events, mitigate psychological distress, and promote communication and well-being. The concept of “art therapy” was introduced by the British artist Adrian Hill in 1942, grounded on his personal experience of a long hospitalization, where artwork alleviated his suffering [1]. Since then, art therapy programs have spread throughout countries and medical disciplines, including psychiatry, neurology, pain management, and oncology [2,3,4,5]. Across different art forms and clinical contexts, increasing evidence suggests that art-based methods support patients and healthcare providers’ psychosocial well-being, agency, and empowerment. Active visual art therapy, in particular, is defined as any type of artistic activity in which patients actively manipulate materials with their hands, such as painting, drawing, sculpting, and craft works [5].

A growing amount of literature has highlighted the effectiveness of AVAT on different patient outcomes. A recent systematic review of 69 randomized controlled trials found that AVAT was associated with improvements in health outcomes, especially in the area of mental health or when treating somatic conditions associated with impaired mental health [5]. Of interest, the majority of the included studies have been conducted in the areas of psychiatric or physical rehabilitation aftercare or for chronic conditions, mainly in outpatient settings or residential facilities. Contrariwise, evidence on the use of AVAT inside the general hospital is still limited, although hospitalization represents a highly stressful and potentially traumatic experience per se and depressive and anxiety symptoms are common in medically hospitalized patients [6,7]. Art therapy has been shown to help inpatients’ unaddressed psychosocial distress and to improve not only the management of physical symptoms but also overall well-being and socialization [8]. AVAT reduced pain and anxiety during acute hospital treatments in adults [9,10] and adolescents [11].

The general hospital poses unique challenges in terms of feasibility, heterogeneity of setting, participants, and underlying medical conditions. At the same time, it represents an area of great interest, need, and potential to improve inpatients’ well-being and quality of life, with significant ethical implications in terms of the humanization of care.

In this regard, the four key principles of biomedical ethics introduced in 1979 by Childress and Beauchamps may represent a useful paradigm to critically evaluate the use of AVAT inside the general hospital, integrating the evidence derived from more traditional psychosocial and physical outcomes [12]. The issues of autonomy, beneficence, non-maleficence, and justice still provide a meaningful conceptual framework to reflect on medical dilemmas and controversial matters in clinical practice.

The present narrative review aims to describe the use of AVAT in the general hospital through the lens of the four core ethical principles of bioethics. Potential implications for future research and clinical application for effective, patient-centered care in the general hospital are discussed.

## 2. Materials and Methods

**Figure 1:**
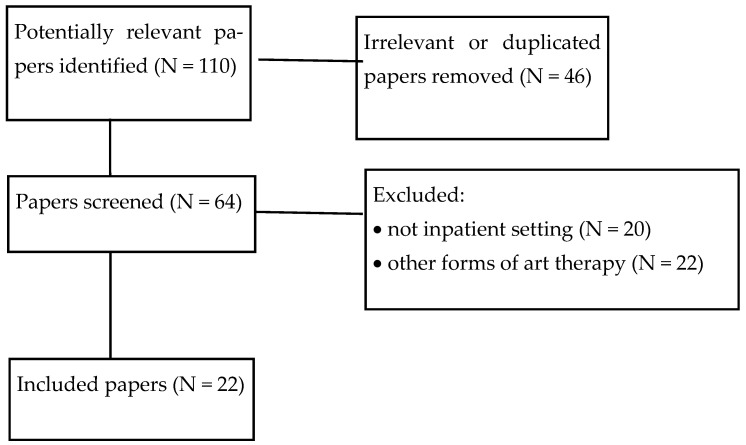
Flow diagram of paper selection process.

A search of the literature was conducted in December 2024 on the Medline and Pubmed databases to identify articles that explored the efficacy of active visual art therapy in hospital settings with the following keywords: visual art therapy; general hospital; inpatients; ethics; psychosocial outcomes; autonomy; beneficence; non-maleficence; and justice. These keywords were used as alternatives (i.e., connected with the logical operator “OR”) in order to reduce the risk of missing relevant papers. Two authors (V.M. and E.L.) independently screened articles by title and abstract to identify relevant papers. The inclusion criteria were: considering the use of AVAT in inpatient settings; having been published between 2004 and 2024; and being written in English or Italian or French. The exclusion criteria were: considering other forms of art therapy (not including AVAT); reporting data regarding outpatients or community samples; and failing to meet inclusion criteria. The selected papers were critically revised, and any disagreement was solved through discussion with two other authors (L.F.P. and M.C.) to reach a consensus. The selection process followed the PRISMA model [13], as shown in Figure 1.

All the co-authors reviewed and discussed the resulting draft to provide a theoretical point of view concerning the main ethical implications of providing AVAT in the general hospital. The final version of the manuscript was then recirculated and approved by all the co-authors.

## 3. Results

### 3.1. Autonomy

The concept of autonomy refers to an individual’s right to self-determination and their ability to make decisions about their medical treatment based on their own values and beliefs. Autonomy is essential for expressing informed consent to medical and surgical treatments. Autonomy encompasses three main aspects: understanding (i.e., the ability to understand what will happen during the intervention and comprehend its expected, possible, or probable beneficial and/or harmful consequences), intentionality (i.e., the willingness to undergo the specific intervention), and non-control (i.e., the absence of external influence or coercion) [14,15]. Moreover, the patient autonomy model needs to consider the impact of the disease on the individual’s capacity for autonomous decisions. The illness itself, with its burden of emotional reactions, especially in the context of hospitalization, may represent a main obstacle to the patient’s autonomy [15].

Although the specific effects of art therapy on the patients’ autonomy have not directly been evaluated, studies suggest that visual art therapy could help patients’ autonomy as it improves self-confidence and enhances communication. Concerning the hospital setting, Brady et al. [10] investigated the role of art therapy within an acute adult admission psychiatric unit using a mixed-method research design involving service users and mental health team members during a 3-month study period. The key elements reported by study participants included developing confidence and improved communication with others, which helped to make sense of their own situation, together with a sense of achievement or renewed self-confidence. The main benefits perceived by the interviewed staff members were reducing social isolation, building confidence, and providing a positive activity with opportunity for self-expression for inpatients. Notwithstanding the limitations of the study in terms of the small sample size and the lack of a control group, these findings support the role of art therapy in enhancing patients’ self-confidence and communication, ultimately leading to improved autonomy in the context of a multidisciplinary adult acute psychiatric service.

With regard to understanding, art could also be used more specifically to help patients comprehend their disease or a particular medical procedure. Using 3D models, drawings, or other forms of visual arts may help doctors better communicate, to patients, information about their condition or the surgery/treatment they need to undergo, hence improving their ability to provide informed consent. In this regard, a scoping review by Traynor et al. [16] analyzed the use of 3D models in enhancing patient–doctor communication in pediatric and adult hospital settings. The review found that, although the use of 3D models appears as a promising method, more research is needed as many of the studies were heterogeneous, and often focused on education rather than communication. Shi and Wang [17] reviewed the use of virtual reality as a form of art therapy to improve health communication. Future research should assess the efficacy of visual reality -art therapy as an intervention to improve communication in general hospital settings.

### 3.2. Beneficence and Non-Maleficence

Beneficence is defined as the responsibility of the healthcare worker to ensure the well-being of patients by only acting in ways that will benefit them. Non-maleficence refers to the idea that healthcare workers should not cause any harm [14]. Both beneficence and non-maleficence are very important ethical aspects to consider when deciding what interventions to administer, keeping in mind the risks versus benefits that may be associated with it.

Recent years witnessed a growing interest in assessing the impact of AVAT in clinical settings on different physical and psychosocial health measures. Joschko et al. [5] conducted a systematic review to assess the use of active visual therapy in improving health outcomes in patients. The authors analyzed 69 randomized controlled trials, assessing 217 outcomes, including psychological distress, self-esteem, quality of life, and social adjustment. The review included approximately 4200 participants, aged between 4 and 96 years, and described a variety of art interventions, mainly focused on drawing/sketching or painting, followed by arts and craft works, sculpting, and coloring in/mandalas. The majority of included research investigated AVAT in the field of mental health (37 studies), neurologic symptoms, and prevention. Art therapy interventions were found to be beneficial in 18% of the outcomes studied, especially in the area of mental health or when treating somatic conditions that may be associated with impaired mental health. However, many of the studies included were of low quality, so more research is needed to assess the true effects of visual art therapy on mental health, focusing specifically on general hospitals.

Mengqin et al. [18] compared the use of mandala art therapy (MAT) and standard preoperative care in a prospective nonrandomized controlled trial involving 126 gynecological cancer patients in a university hospital in China. Participants were assigned to either receive the MAT program or standard perioperative care. The interventions took place before surgery following preoperative preparation, on postoperative day 2 or day 3 and on the discharge day. Results suggest that mandala art therapy helps reduce stress (with a significant reduction in the Visual Analog Scale for Stress) and anxiety (with a significant reduction in the State-Trait Anxiety Inventory score), before undergoing surgery. Additionally, perceived waiting time before surgery and blood pressure values were both significantly improved in the intervention group compared to standard of care. Of note, there were no statistically significant differences between groups regarding hope and self-acceptance assessed by the Herth Hope Index (HHI) and Self-acceptance Questionnaire (SAQ). One possible explanation regards the limited duration of the MAT intervention in the perioperative period, compared to outpatient settings. In this study, the sessions were limited by the length of stay of perioperative patients, suggesting that follow-up with MAT services once discharged may be necessary. Although the study presents methodological caveats—mainly the single-center nonrandomized design, affecting the generalizability of data—it provides a valuable contribution to a deeper understanding of the potential benefits of AVAT for gynecological cancer patients during the perioperative period in the hospital setting, also in terms of feasibility and acceptance.

Shella [9] investigated the impact of a single art therapy session administered at bedside in a sample of 195 study participants admitted to a general hospital through a retrospective chart review. Study participants were engaged in a 50 min art therapy session and were asked to rate their perception of mood, anxiety, and pain before and after the intervention. Types of media included paints, beads, and mixed media. The authors highlight that a strong emphasis was placed on patients’ choice in terms of materials chosen and the decision to participate. Diagnoses included malignancies, neurological diseases, gastrointestinal conditions, cardiovascular diseases, transplants, and patients undergoing surgical and orthopedic procedures. The authors found significant improvements in pain control, mood, and anxiety, assessed through a self-administered tool (i.e., the Roger’s Happy Sad face), following the art intervention. Important limitations of the study include the retrospective design, the lack of a control group, and the immediate evaluation of the effects of art therapy in a single session. However, the findings provide a useful insight in terms of the benefits and feasibility of art therapy at bedside in an inpatient, acute-care setting.

Mollaogl˘u et al. [19] investigated the effects of art therapy in breast cancer hospital patients enrolled in a randomized controlled trial. The study compared 30 women in the intervention group (marbling and ney music) to 30 women in the control group (no intervention). The intervention group showed significantly lower pain levels, less anxiety, better quality of life, and less nausea, as assessed by the Pain Intensity Measurement Visual Analog Scale (VAS), Beck Anxiety Inventory (BAI), Functional Assessment of Cancer Therapy Scale General (FACT-G), and the Rhodes Index of Nausea, Vomiting, and Retching (RINVR) respectively.

Zahmatkesh et al. [20] investigated the impact of four sessions of art therapy on the experience of grief and the quality of life of women who experienced abortion or stillbirth in the previous 6 weeks, referring to the maternity wards of two Iranian hospitals [20]. The trial involved 60 women, randomly assigned to the intervention group (N = 30), including active visual art therapy, and the control group (N = 30), in which routine care was performed. Art therapy was associated with a significant improvement in the mean total quality of life score assessed by the World Health Organization quality of life questionnaire, short version 26. Also, all physical, psychological, social, and environmental dimensions of quality of life improved in the art therapy group compared to the control group eight weeks after the intervention.

An important area of application of visual art therapy is its use with inpatient children and adolescents. As cited in Hen [21], previous studies found long-term art therapy with hospitalized children with cancer, chronic diseases, or mental illness to be effective [22,23] and contribute to their healing process [24,25]. Ferrari et al. recently provided a description of creative and artistic activities and laboratories, including active visual art therapy, developed for adolescents and young adults with cancer promoted by the Italian Association for Pediatric Hematology-Oncology (AIEOP) adolescents working group [26]. The authors reported different experiences from 10 hospitals in Italy and discussed the main goals of creative projects, as a way for patients to share their difficulties with peers, to find a sense of light-heartedness during their hospital stays, and regain a sense of future, working on projects expected to take several months. Of note, the authors underline the value of dedicated spaces and opportunities for socializing and recreation, but also the need to preserve privacy and provide respect and protection for cancer patients, stating how art projects should be professionally organized and realized within the hospital [26].

In a different context, recent research by Bifano and Tsze [11] found that visual art therapy could reduce levels of pain and anxiety in children in the emergency room. The study included a convenience sample of 50 patients presenting with a painful condition to a tertiary-care children’s hospital emergency department. The mean duration of art therapy was 34.7 min. Mean baseline pain assessed by the Verbal Numerical Rating Scale (scored 0–10) was 6.2 and decreased by 23.2% and 28.6% immediately after and 1 h after art therapy completion, respectively. Mean baseline anxiety assessed by the State-Trait Anxiety Inventory short form was 48 (moderate) and decreased to 38 (low) and 43 (moderate) at the same time points. Patients reported feelings of relaxation, decreased pain intensity, and/or empowerment. The study presents important methodological limitations, in terms of the absence of a control group, the administration of analgesic as a potential confounder with regard to the impact of art therapy on perceived pain, and the risk of observer and/or participant bias. However, the potential benefits in reducing anxiety and improving the qualitative experience of adolescents with painful conditions in the high-stress environment of the emergency department deserve attention. Moreover, participants reported that art therapy gave them a sense of control and empowerment, suggesting the role of art therapy in promoting autonomy.

Additionally, Versitano et al. [27] found that visual art therapy reduces the use of restrictive practices in children in the acute inpatient child and adolescent mental health service unit. The rate, frequency, duration, and total number of incidents of seclusion, the frequency and total number of incidents of physical restraint, and the rate, frequency, and total number of incidents of intramuscular sedation showed a statistically significant reduction during phases of art therapy service provision over a 6-year period. The art therapy service in the study was a fixed component of the therapeutic group program. Boredom, which can be a result of occupational deprivation commonly occurring in restrictive environments such as a locked unit, often precipitates increased agitation or aggression, potentially leading to restrictive practices. Art therapy can effectively engage young people, support emotional regulation, and promote social interaction, preventing increased aggression or agitation.

Of note, implementing AVAT in general hospitals can help humanize them and further aid in the recovery of patients. Hospitals can often look sterile and impersonal to patients, which can be detrimental to their well-being and recovery. Visual art has the potential to beautify hospitals, and in doing so make them more relatable and more humane. Lankston et al. [28] discussed the role of visual art displayed in hospitals in promoting not only patients’ well-being but also that of healthcare workers and other service users. The authors evaluate and discuss the visual art of three Scottish hospitals and explain that specific colors, such as blue and green, and paintings of nature may be especially calming. Although this study did not imply an active intervention, it provides a broader reflection and some suggestions with regard to the potential benefits of visual art inside the hospital in terms of promoting individual and group well-being, supporting autonomy. More recently, a systematic review by Law et al. (2021) [29] found that viewing visual artworks in hospitals may reduce stress in patients, evidencing biological benefits such as a decrease in systolic blood pressure and decreased heart rate.

Art therapy is beneficial not only for patients in hospitals but also for healthcare workers. Working in hospital settings, although rewarding, can often cause psychological distress and burnout. Moss et al. (2022) investigated the feasibility, acceptability, and psychosocial outcomes of four creative art therapy (CAT) interventions, including active visual art therapy, in a cohort of 146 healthcare professionals during the COVID-19 pandemic [30]. Participants included nurses (52%), physicians, and other professionals (i.e., behavioral therapists, social workers) practicing in a hospital setting for at least 20 h/week experiencing significant burnout symptoms assessed by the Maslach Burnout Inventory. Participants were randomized to one of the four CAT intervention groups, which met in person for a total of 12 consecutive 90 min weekly sessions. The program proved to be feasible and acceptable in terms of attendance and clients’ satisfaction assessed by the Client Satisfaction Questionnaire (CSQ-8). Participants randomized to the intervention showed significant improvements in anxiety and depression scores, total post-traumatic stress disorder score, burnout scores, and turnover intention. According to a further study by Avallone Mantelli et al. [31], the intervention group showed sustained improvements in anxiety, depression, and total PTSD score compared to baseline at 1-year follow-up. Moreover, compared to control participants, the intervention group showed improvements at one year in anxiety, depression, positive affect, and negative affect using the Positive and Negative Affect Scale (PANAS). Of interest, Moss et al. underlined the limited costs of the CAT interventions and the absence of safety concerns: the intervention’s risk or cost-to-benefit ratio for improving healthcare professional’s psychological distress is therefore potentially large, with potential indirect benefits to assisted persons too.

Recent meta-analytic evidence [32] explored the use of art therapy in improving mental health in nurses. The study found that art therapy helped reduce anxiety, with a significant reduction in the Self-Rating Anxiety Scale scores. Depression was also significantly lower in nurses undergoing art therapy compared to the control group. Similarly, pressure was significantly reduced in the intervention groups compared to the controls. The study also found that positive copying style was higher in the intervention group. Although the meta-analysis included overall high-quality studies, it examined different modalities of art therapy, such as painting, music, psychodrama, drama, and sculpture therapy, making it harder to assess the specific effects of visual art therapy. Additionally, the authors focused on nurses in different clinical settings, making it hard to understand the efficacy of art therapy specifically in general hospitals.

Ong et al. [33] analyzed the effectiveness of art therapy (creating collages and boxes) in improving empathy and well-being in junior doctors during their palliative care rotations. The study found that participants enjoyed the experience but lacked pre-existing knowledge of humanities and their relations to medical training. The participants mentioned improved empathy and connecting with both patients and fellow doctors, but at the same time, concerns about the program being implemented at their hospitals due to concerns about having a safe space and sharing feelings with peers.

### 3.3. Justice

Justice refers to the need for appropriate, fair, and equitable distribution of medical resources [14].

In a systematic review evaluating the clinical benefits and cost-effectiveness of group art therapy for people with non-psychotic mental disorders, art therapy appeared to be cost-effective compared to waiting list, but the strength of the results was limited by methodological issues like the substantial heterogeneity in the patient clinical profiles and the low quality of the included trials [34]. Notwithstanding these caveats, these findings are relevant to the implementation of art therapy in the general hospital, given the fact that non-psychotic mental disorders, including depressive and anxiety symptoms, represents the most common comorbid psychiatric issues in hospitalized patients across different specialties [6,7].

Of interest, in a study investigating a single brief art therapy intervention at bedside during acute hospital treatment, art therapy proved to be a safe and cost-effective adjunct intervention to traditional medical management [9].

Equitable access to healthcare is a fundamental ethical issue. In this regard, the systematic review by Joshko et al. [5] underlined huge differences across countries in AVAT research, with the vast majority of studies conducted in the USA (23) and the UK (5). This discrepancy may reflect different factors influencing research priorities but also calls for the need to promote equitable healthcare access by extending the benefits of AVAT to diverse populations.

## 4. Discussion

The presence of art therapy programs in healthcare has become widespread in the 21st century. Current evidence supports the effectiveness of visual art therapy on children and adult patients’ outcomes across different disciplines, particularly in the area of mental health [5]. Additionally, studies suggest that art therapy may be effective for healthcare workers to ameliorate their communication and avoid burnout. Improving patient–doctor communication can be especially beneficial for patients—children and adults—who have difficulties in using traditional forms of verbal communication, as required by more standard forms of treatment for mental health problems. Art therapies can provide an alternative means of expression to help patients understand, make sense of, and cope with their distress [10,34].

This narrative review aimed to reflect on the use of AVAT from an ethical perspective in the context of the general hospital, as summarized in Table 1. During his Nobel Lecture in Literature in 1987, Joseph Brodsky commented that “On the whole, every new aesthetic reality makes man’s ethical reality more precise. For aesthetics is the mother of ethics” [35]. This quote exemplifies how ethics and the arts are intertwined. AVAT may support patients’ autonomy, contribute to their beneficence with no or limited risks, and represent an equitable and cost-effective treatment in the general hospital. The recent literature has explored the need for humanization of care, a concept that extends patient-centered care introduced by Balint in 1969 to involve all stakeholders—patients, caregivers, and healthcare professionals—and their interactions. A systematic review by Busch et al. [36] identified an empathetic and respectful approach to patients, sufficient human and material resources in healthcare institutions, and a balanced workload for healthcare providers as the key elements for establishing meaningful, mutually beneficial relationships with patients and delivering humanized care [37]. Research has shown how AVAT may contribute to all these aspects in the general hospital.

Previous authors underlined the importance of art therapy in restoring hope, fun, and the enjoyment and value of creative play in mental health as a means to overcome psychological stiffness and become involved in artwork [10,37]. These observations recall Donald Winnicott’s “Squiggle Game”, and the use of art and doodling to break boundaries between a patient and professional to narrate a story through a simple squiggle [1]. Research suggests that the active manipulation of art materials elicits interoceptive and somatosensory processing that may decrease pain and anxiety by amplifying positive emotional reactions and feelings, with potential clinical applications for treating painful conditions and managing procedural pain and anxiety in the general hospital [11,38].

General hospitals are particular settings, as they can include different staff, patients, and clinical conditions. Therefore, it is not easy to conduct homogeneous studies and randomized controlled trials, especially in the context of ethical aspects and the specific impact that art has on them.

Methodological challenges faced by the majority of studies exploring AVAT in the hospital setting include the small sample size, heterogeneity of study participants, and lack of a control group in single sites studies [9,10]. Research needs to balance the potential tension between scientific requirements in terms of outcome measures using standardized validated reproducible instruments and the unique experience of art therapy, implying a variety of theories and methods.

Joshko et al. underlined the need for established guidelines to enhance research consistency and comparability, including standards for reporting AVAT and suitable control interventions [5]. This is crucial, from a scientific, clinical, and ethical perspective, to providing a solid foundation for evidence-based decision making and allocation of resources.

Several unique ethical considerations emerge when working at the intersection between art practice and the individual experiences related to the disease condition and in the context of hospitalization.

With regard to beneficence and non-maleficence, safety is a major concern. Special attention is required in the general hospital in terms of materials used, hygiene, and sterile environments. Of note, none of the reviewed studies reported adverse events in this sense. From a broader psychosocial perspective, safety may refer also to the way art interventions are delivered to vulnerable individuals or groups, including inpatients (Ferrari et al., 2024, Pavarini, 2021). In this regard, art therapy is underpinned by its own professional code of ethics and conduct, with shared central values guiding practice, including respect, competence, responsibility, and integrity [26,39].

## 5. Conclusions

Art-based therapy could be a widely accessible and fairly inexpensive therapeutic approach to improve patient–staff communication and well-being.

We aimed to provide a critical review of the available data on the use of AVAT in the general hospital. The value of art interventions is often described and well recognized in non-scientific journals, and a number of clinical experiences seem to support this view; however, to reach an established scientific status, AVAT still lacks sufficiently solid proofs supporting its utility in the majority of the contexts where it has been applied. Further methodologically sound research is particularly needed to understand the effectiveness and real feasibility of AVAT in general hospitals.

Practical recommendations for further research include to identify key clinical and psychosocial measurable outcomes to be systematically investigated through validated measures. Research should expand to include different chronic conditions, including neurological and cardiovascular diseases, and transplant medicine. Acute settings represent a major challenge for clinical application and research, in terms of feasibility, acceptability, and beneficence/non maleficence ratio. In this regard, data on the effectiveness of creative art therapy in ameliorating burnout and psychological distress in healthcare providers support the implementation of these practices for the staff working in emergency units, which in turn may benefit patients and their family members. A more in-depth awareness of the ethical aspects implied in using AVAT supports the implementation of art interventions to promote patient-centered and humanized care in the general hospital.

## Figures and Tables

**Table 1:** Ethical issues related to the main outcomes of active visual art therapy (AVAT) in hospitalized patients.

Author	Study Design	Main Ethical Issues Implied	Quantitative Measures	Investigated Domains/Main Outcomes	Relevance
Bifano and Tsze, 2024 [11]	Prospective pilot study	Beneficence	Verbal Numerical Rating Scale (VNRS); short-form six-item State-Trait Anxiety Inventory (STAI:Y-6)	One single session of AVAT was associated with a reduction in pain and anxiety in a sample of 50 patients presenting with a painful condition to a tertiary-care children’s hospital emergency department	*
Brady et al., 2017 [10]	Mixed-method design	Autonomy, Beneficence, Justice	Ad hoc quantitative questionnaire	Art therapy was reported to reduce patients’social isolation, help building confidence and promoting self expression according to a survey of 35 staff members and 11 service users in an acute adult admission psychiatric unit	**
Mengqin et al., 2024 [18]	Prospective, non-randomized, controlled trial	Beneficence	Visual Analog Scale for Stress (VASS); State-Trait Anxiety Inventory-state scale (STAI-S); Herth Hope Index (HHI); Self-acceptance Questionnaire (SAQ)	Mandala art therapy reduced perceived stress, anxiety, and blood pressure in gynecological cancer patients before surgery compared to standard care	**
Mollaogl˘u et al., 2024 [19]	RCT	Beneficence	Pain Intensity Measurement Visual Analog Scale (VAS); Beck Anxiety Inventory (BAI); Functional Assessment of Cancer Therapy Scale—General (FACT-G); Rhodes Index of Nausea, Vomiting, and Retching (RINVR)	The art intervention based on marbling and ney music was associated with lower perceived pain, lower anxiety, better quality of life, and less nausea breast cancer hospital patients	**
Shella et al., 2017 [9]	Retrospective chart review	Beneficence	Roger’s Happy Sad face scale	A single session of AVAT at bedside improved mood, anxiety, and pain in inpatients admitted to a general hospital	*
Versitano et al., 2024 [27]	Naturalistic observational retrospective study	Beneficence, Non-maleficence	Rate (events per 1000 occupied bed days); frequency, duration, and number of incidents of restrictive practice; rate, frequency, and number of incidents of intramuscular injected sedation, oral as-needed medication use, and absconding incidents occurring in conjunction with an episode of seclusion or restraint	The provision of an art therapy program was associated with a reduction in the number of incidents of seclusion, physical restraint, and intramuscular sedation in one acute inpatient child and adolescent mental health service unit compared to periods in which the intervention was not available	**
Zahmatkesh et al., 2024 [20]	RCT	Beneficence	Perinatal Grief Scale (PGF); World Health Organization quality of life questionnaire (WHOQOL-BREF)	Art therapy was associated with a significant improvement in quality of life in women who recently experienced abortion or stillbirth referring to the maternity unit of two general hospitals	**

RCT: randomized controlled trial; relevance: * small sample size and/or methodologically weak; ** fair; *** relevant.

## Data Availability

Not applicable.
